# Endoscopic submucosal dissection using the tunneling method for early gastric cancer occupying the entire fornix

**DOI:** 10.1055/a-2321-9626

**Published:** 2024-05-29

**Authors:** Yohei Yabuuchi, Soichiro Nagao, Shuko Morita, Tetsuro Inokuma

**Affiliations:** 1 26330 Department of Gastroenterology, Kobe City Medical Center General Hospital, Kobe, Japan

The location of the fornix is challenging for endoscopic submucosal dissection (ESD) because of its difficult access, tendency to face vertically, and submersion in the gastric fluid and blood. To overcome these issues, the use of a multi-bending scope, multiple clip-line tractions, and right lateral position have been reported 1 2 3 . However, even with these strategies, treating lesions occupying the entire fornix remains challenging because adverse events such as perforation have been reported 4 . We describe a successful ESD for early gastric cancer occupying the entire fornix using the tunneling method, allowing us to approach the most difficult-to-reach area from a tangential direction and achieve resection without adverse events.

An 82-year-old woman with a history of rheumatoid arthritis, hypertension, and hyperlipidemia was referred to our hospital following the detection of a lesion in the fornix during an esophagogastroduodenoscopy performed previously. Endoscopic examination at our institution revealed a large, superficially elevated lesion with a small nodule occupying the entire fornix, which was diagnosed as early gastric cancer ( Fig. 1 , Fig. 2 ). No obvious signs of submucosal invasion were observed and endoscopic resection was considered.

**Fig. 1:**
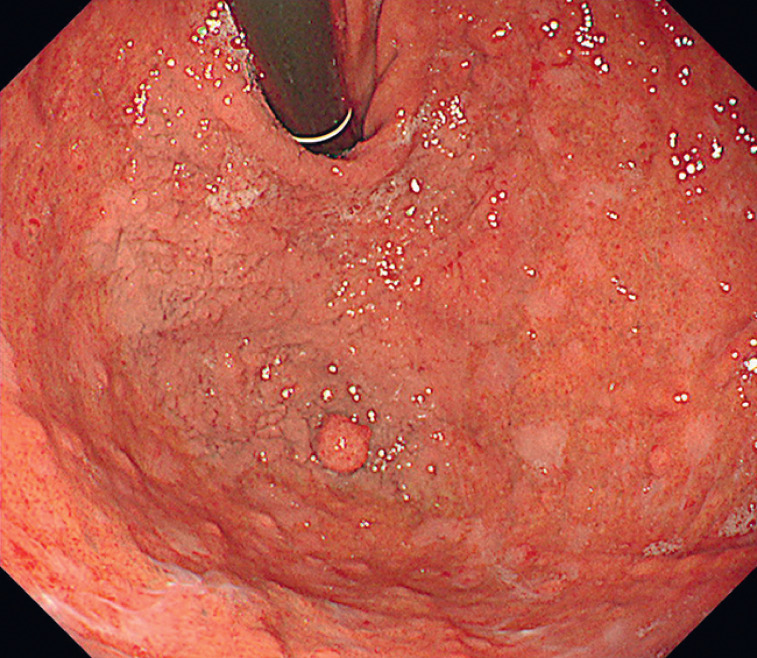
A large, superficially elevated lesion with a small nodule occupying the whole fornix.

**Fig. 2:**
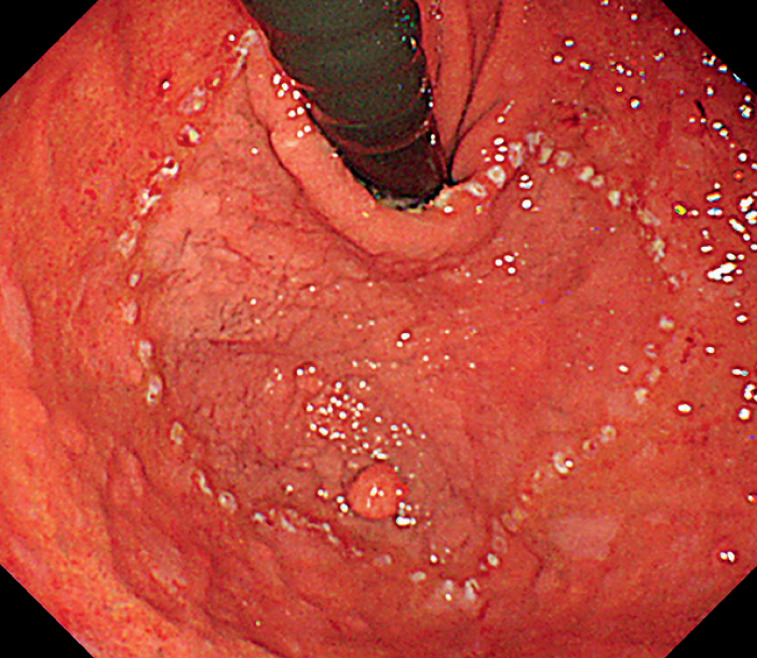
A large, superficially elevated lesion with a small nodule occupying the whole fornix after marking.

ESD was performed using a therapeutic scope (GIF-H290T; Olympus, Tokyo, Japan) and a multi-bending endoscope (GIF-2TQ260M; Olympus) with an ITknife nano (KD-612Q; Olympus) and a Clutch Cutter (DP2618DT-35; Fujifilm, Tokyo, Japan). We first made a mucosal incision and trimmed the side of the greater curvature in the right lateral position to create an endpoint. Subsequently, the tunnel entrance was made through the cardia, followed by the creation of the submucosal tunnel ( Fig. 3 ). After a circumferential incision was created, a submucosal dissection was performed in retroflexion using multiple dental floss clip tractions 5 , resulting in en bloc resection with a specimen size of 100 × 83 mm without adverse events ( Fig. 4 , Fig. 5 ; Video 1 ). A well-differentiated intramucosal adenocarcinoma measuring 75 × 68 mm without ulceration and negative margins was diagnosed, indicating curative resection.

**Fig. 3:**
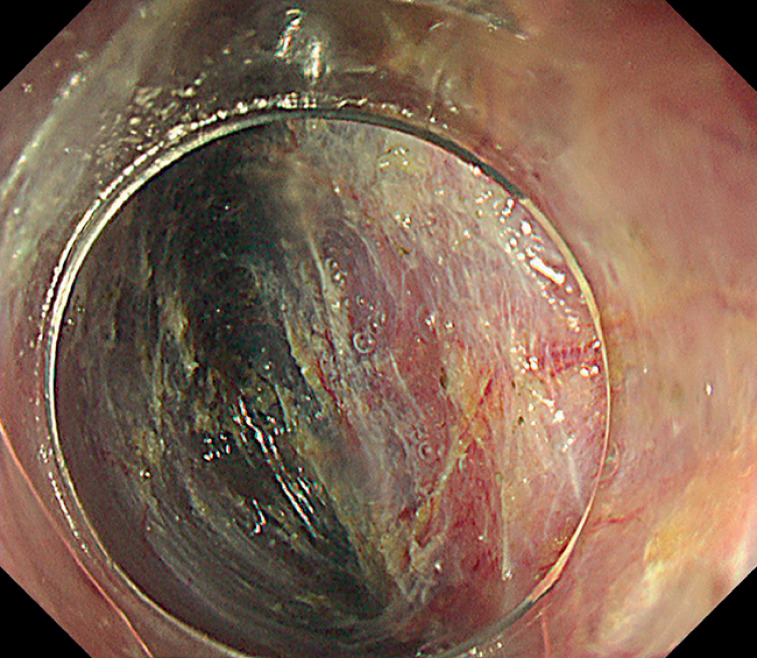
Creating the submucosal tunnel.

**Fig. 4:**
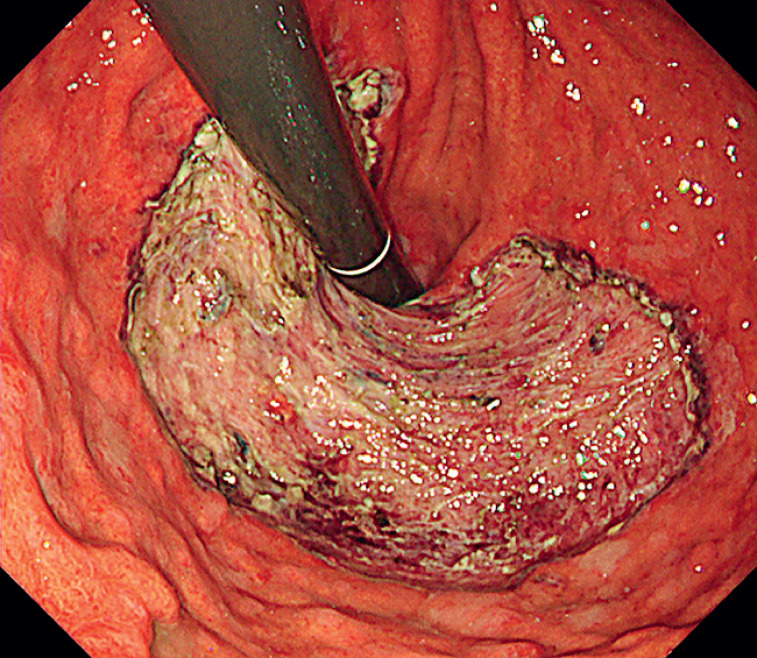
Mucosal defect after endoscopic submucosal dissection.

**Fig. 5:**
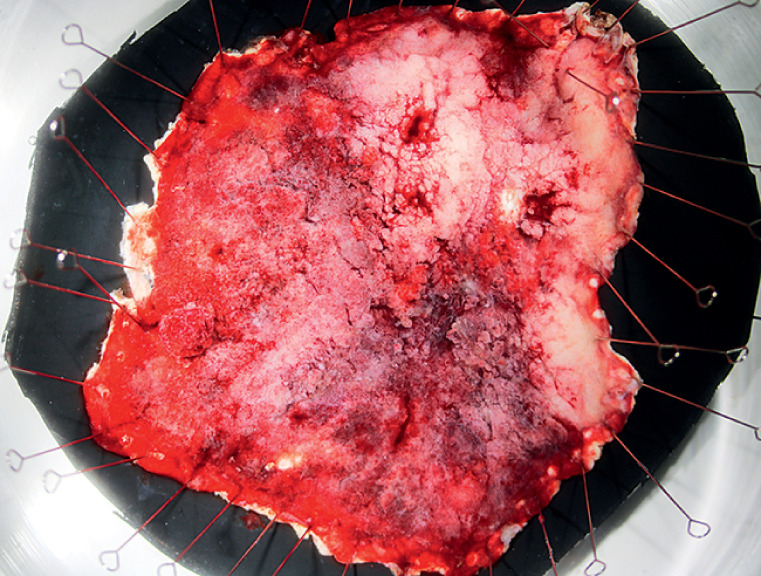
En bloc resection was achieved.

Endoscopic submucosal dissection using the tunneling method for early gastric cancer occupying the entire fornix.Video 1

Endoscopy_UCTN_Code_TTT_1AO_2AG_3AD

## Notes

Endoscopy E-Videos https://eref.thieme.de/e-videos
*E-Videos* is an open access online section of the journal *Endoscopy* , reporting on interesting cases and new techniques in gastroenterological endoscopy. All papers include a high-quality video and are published with a Creative Commons CC-BY license. Endoscopy E-Videos qualify for HINARI discounts and waivers and eligibility is automatically checked during the submission process. We grant 100% waivers to articles whose corresponding authors are based in Group A countries and 50% waivers to those who are based in Group B countries as classified by Research4Life (see: https://www.research4life.org/access/eligibility/ ). This section has its own submission website at https://mc.manuscriptcentral.com/e-videos .
